# Untargeted LC–MS/MS Metabolite Profiling of Wild, Field-Domesticated and Tissue-Cultured Rhizomes of the Endangered Medicinal Fern *Cibotium barometz*

**DOI:** 10.3390/life16091435

**Published:** 2026-08-28

**Authors:** Yezhen Su, Guole Qin, Changqin Zhou, Delong Guan, Jing Song

**Affiliations:** 1 Guangxi Key Laboratory of Sericulture Ecology and Intelligent Technology Application, Guangxi Collaborative Innovation Center of Modern Sericulture and Silk, Guangxi Colleges Universities Key Laboratory of Exploitation and Utilization of Microbial and Botanical Resources, School of Chemistry and Bioengineering, Hechi University, Hechi 546300, China; 2022101882@hcnu.edu.cn (Y.S.); 06032@hcnu.edu.cn (G.Q.); 2023660006@hcnu.edu.cn (D.G.); 2 Zhongnong Qiaosheng (Fangchenggang) Traditional Chinese Medicine Technology Co., Ltd., Fangchenggang 538037, China

**Keywords:** *Cibotium barometz*, domestication, flavonoids, herbal authentication, LC–MS/MS, medicinal fern, organic acids, phenolic acids, tissue culture, untargeted metabolomics

## Abstract

*Cibotium barometz* (Gouji) is a widely prescribed traditional medicine whose supply still depends on wild-harvested rhizomes of this CITES-listed medicinal tree fern. We asked whether field domestication or tissue culture reproduces its chemical profile. Wild-collected (WP), field-domesticated (DM) and tissue-cultured (ZP) rhizomes (*n* = 3) were profiled by untargeted LC-MS/MS. Of 2828 annotated features, 2787 (98.6%) were detected in all three materials and fewer than ten were group-exclusive, indicating that differences are quantitative rather than qualitative, although this overlap partly reflects gap filling and detection limits. Replicate-level distances to the WP centroid were metric-dependent: correlation- and magnitude-based measures ranked DM closer (1 − Pearson 0.290 vs. 0.371), whereas abundance-weighted Bray–Curtis favoured ZP (0.398 vs. 0.459), consistent with PCA showing both substitutes displaced from WP along independent axes. Within nine literature-prioritized classes, ZP retained 93% of the WP response and an essentially identical organic-acid core (log_2_FC = −0.01), whereas DM retained 63%, losing organic acids, alkaloids, nucleosides and organosulfur compounds and gaining flavonoids and pterosins. N6-hydroxymethyladenosine, crotonoside and modified nucleosides are proposed as candidate authentication markers requiring validation with authentic standards. Chemical similarity alone does not establish therapeutic equivalence.

## 1. Introduction

The rhizome of *Cibotium barometz* (L.) J. Sm. (Cibotiaceae), known as Gouji or Rhizoma Cibotii, is officially recorded in the Chinese Pharmacopeia and has been prescribed for centuries to tonify the liver and kidney, strengthen the sinews and bones, and relieve lumbago and rheumatic pain [1]. Contemporary pharmacological work attributes these activities to a chemically heterogeneous set of specialized metabolites, including phenolic acids (protocatechuic acid and aldehyde, caffeic and chlorogenic acids), flavonoids and proanthocyanidins, pterosin-type sesquiterpenoids, phytoecdysteroids, and phytosterols, which have shown antioxidant, anti-inflammatory, osteogenic and anti-osteoporotic effects in vitro and in animal models [1,2,3,4]. Consequently, the therapeutic value of Gouji is best understood not as the property of a single marker compound but as an emergent property of its metabolite profiles [1,5,6]. Recent phytochemical surveys have further expanded the reported constituent list of the rhizome, including new pterosin-type sesquiterpenoids and phenolic glycosides [1,3].

Persistent demand has, however, outstripped natural regeneration. *C. barometz* is a slow-growing tree fern with limited spore establishment, and decades of destructive rhizome harvesting have fragmented wild populations across southern China and Southeast Asia, leading to its listing in CITES Appendix II and in national protection catalogues [7]. Despite this status, material entering the herbal supply chain is still predominantly wild-collected, creating a conflict between conservation obligations and pharmaceutical supply that can only be resolved by cultivated substitutes [1].

Two substitution routes are currently pursued. Field domestication transplants and propagates wild germplasm under managed agronomic conditions, retaining a broadly natural soil, light and microbial environment. Micropropagation through spore- or gametophyte-derived tissue culture offers far higher multiplication rates, pathogen-free planting stock, and independence from wild collection, and has been established for several *Cibotium* lineages [1,6,8]. Yet both routes alter the environmental cues that shape specialized metabolism. Domestication typically relaxes abiotic and biotic stress and may therefore reduce defence-related phenylpropanoid and terpenoid accumulation, whereas in vitro culture imposes an entirely artificial regime—heterotrophic sucrose supply, exogenous phytohormones, low light and high humidity—that is well documented to reprogram secondary metabolism, sometimes suppressing and sometimes strongly eliciting individual pathways [9,10]. Which outcome prevails in *C. barometz*, and whether it compromises or enhances pharmacological quality, has not been tested.

Metabolomics provides a direct route to this question, because it measures the chemical phenotype that ultimately determines herbal efficacy rather than proxies such as biomass or growth rate [11]. Untargeted LC–high-resolution-MS/MS combines broad, hypothesis-free coverage of primary and specialized metabolites with accurate-mass and fragmentation-based annotation, and has been used to compare cultivated substitutes with wild medicinal plants including *Dendrobium*, *Panax* and *Paris* species [12,13,14]. It is, however, inherently semi-quantitative: peak areas reflect both molar abundance and compound-specific ionization efficiency, so profiles can be compared between samples for a given feature but not summed into absolute chemical quantities. Comparable data for medicinal ferns remain scarce, and no study has evaluated tissue culture and domestication of *C. barometz* side by side against a wild reference.

Here we profiled wild-collected (WP), field-domesticated (DM) and tissue-cultured (ZP) *C. barometz* rhizomes by untargeted LC–MS/MS, with pooled QC samples used to verify analytical stability. The study was designed as an exploratory chemical comparison with three aims: to delimit, within the annotated metabolome, the subset of compounds whose structural classes are documented as bioactive in *C. barometz* or related ferns; to test, using correlation-, magnitude- and abundance-weighted distance criteria applied to individual biological replicates, which propagation system yields a profile closer to wild material both overall and within that subset; and to identify individual compounds that differ consistently between wild and propagated rhizomes and could therefore serve as authentication markers. We emphasize from the outset that chemical similarity is a necessary but not sufficient condition for pharmacological interchangeability; the framework proposed here is intended to prioritize materials and markers for subsequent bioassay- and standard-based validation, not to certify substitution.

## 2. Materials and Methods

### 2.1. Plant Material, Propagation Systems and Experimental Design

All plant material was collected in March 2026 from a single large-scale *Cibotium barometz* (L.) J. Sm. cultivation base and the immediately adjacent reserve forest in Fangchenggang, Guangxi Zhuang Autonomous Region, China (21.7 deg N, 108.4 deg E; lateritic red soil, pH 4.5–5.5). Sampling from a single site ensured a common climate, soil parent material and management history. Species identity was verified by Prof. Guole Qin (plant taxonomist, School of Chemistry and Bioengineering, Hechi University) using the treatment of Cibotiaceae in Flora of China, and voucher specimens (accession numbers HCNU-CB-2026-001 to HCNU-CB-2026-003 for WP, DM and ZP, respectively) were deposited in the Herb lab of Hechi University, Hechi, China. Collection of wild material from the reserve forest was not limited; cultivated and tissue-cultured material was supplied by the base operator, Zhongnong Qiaosheng (Fangchenggang) Traditional Chinese Medicine Technology Co., Ltd. (Fangchenggang, China) No CITES export was involved.

Three production systems were compared, each with three independent biological replicates (*n* = 3; nine samples in total). **Wild-collected plants (WP)** were naturally regenerated individuals from the shaded, humus-rich floor of the reserve forest (canopy closure ≈ 0.8; midday understorey PPFD 20–80 µmol m^−2^ s^−1^), receiving no management, irrigation or fertilization; as age could not be determined non-destructively, plants were matched for rhizome fresh weight (≈0.8–1.5 kg) and a fully expanded frond crown, the stage at which wild Gouji is conventionally harvested. **Field-domesticated plants (DM)** were wild-derived germplasm grown for three years in a shade house (70% shade net; 25–30 °C; RH 70–85%) on lateritic red soil amended with decomposed cattle manure at 1.0 m × 1.0 m spacing, drip-irrigated to 60–70% of field capacity and top-dressed twice yearly (N:P_2_O_5_:K_2_O = 15:15:15; 300 kg ha^−1^, March and August), with no pesticide applied in the 12 months before harvest at the commercial stage. **Tissue-cultured plantlets (ZP)** were regenerated from rhizome apex explants (2–3 mm) of spore-derived gametophyte cultures of the same provenance; after surface sterilization (75% ethanol, 30 s; 2% NaClO, 10 min; five sterile-water rinses), shoots were multiplied on MS medium with 2.0 mg L^−1^ 6-benzylaminopurine, 0.2 mg L^−1^ NAA, 30 g L^−1^ sucrose and 7 g L^−1^ agar (pH 5.8) at 25 ± 2 °C under a 12 h photoperiod (40 µmol m^−2^ s^−1^), subcultured every 30 d for four cycles, rooted on half-strength MS with 0.5 mg L^−1^ NAA and 20 g L^−1^ sucrose for 45 d, then transplanted to peat:perlite (3:1, *v*/*v*) and acclimatized for six months under the same shading and irrigation regime as DM plants before harvest.

The three groups differ not only in propagation route but also in plant age (undetermined for WP, three years for DM, approximately one year from explant for ZP) and, for WP, in growing environment (forest floor versus shade house). This confounding is intrinsic to the comparison, because the material examined here is precisely the material that is, or would be, commercially available at its normal harvest stage. Each biological replicate consisted of pooled rhizomes from three to five individuals of comparable size and developmental stage, so as to average out plant-to-plant variation. Rhizomes were cleaned of soil and golden trichomes, rinsed with deionized water, blotted dry, frozen immediately in liquid nitrogen in the field, transported on dry ice and stored at −80 °C. Frozen tissue was lyophilised, ground to a fine powder in a ball mill (MM 400, Retsch, Haan, Germany) at 30 Hz for 90 s, and stored at −80 °C until extraction.

### 2.2. Metabolite Extraction and LC–MS/MS Analysis

Untargeted metabolomic profiling was performed by Shanghai Personal Biotechnology Co., Ltd. (Personalbio, Shanghai, China). A standardized protein-precipitation workflow was used. Briefly, 100 mg of lyophilised rhizome powder was weighed into a 2 mL centrifuge tube, and 1000 µL of pre-chilled extraction solvent (methanol:acetonitrile:water, 2:2:1, *v*/*v*/*v*, −20 °C) containing internal standards was added. Samples were vortexed for 60 s, homogenized in a ball mill at 45 Hz for 4 min, and sonicated in an ice-water bath for 30 min. Proteins and macromolecules were precipitated at −20 °C for 1 h, after which samples were centrifuged at 12,000× *g* for 15 min at 4 °C. The supernatant was carefully transferred to a fresh tube and evaporated to dryness in a vacuum concentrator. The dried residue was reconstituted in 200 µL of acetonitrile:water (1:1, *v*/*v*), vortexed, sonicated in an ice-water bath for 10 min, and centrifuged again at 12,000× *g* for 15 min at 4 °C. The clarified supernatant was transferred to autosampler vials for LC–MS/MS analysis. Quality control (QC) samples were prepared by pooling equal aliquots (20 µL) of every experimental sample, and were extracted and analyzed using the identical procedure. Solvent blanks were prepared in parallel to identify background and carry-over features.

Chromatographic separation was performed on a Vanquish UHPLC system (Thermo Fisher Scientific, Waltham, MA, USA) fitted with an ACQUITY UPLC HSS T3 column (100 mm × 2.1 mm, 1.8 µm; Waters, Milford, MA, USA) maintained at 40 °C. The autosampler was held at 4 °C and the injection volume was 2 µL. The flow rate was 0.3 mL min^−1^. For the positive ion mode, mobile phase A was 0.1% formic acid in water and mobile phase B was 0.1% formic acid in acetonitrile; for the negative ion mode, mobile phase A was 5 mM ammonium acetate in water and B was acetonitrile. The gradient was: 0–1 min, 2% B; 1–9 min, 2–98% B; 9–12 min, 98% B; 12–12.1 min, 98–2% B; and 12.1–15 min, 2% B for re-equilibration.

Mass spectrometry was carried out on a Orbitrap Exploris 120 mass spectrometer (Thermo Fisher Scientific) equipped with a heated electrospray ionization source, operated independently in positive and negative ion modes to maximize metabolome coverage (global profiling). Operation process was performed by the Shanghai Personal Biotechnology Co., Ltd., Shanghai, China. Samples were injected in a randomized order to avoid batch- and run-order artefacts. One QC injection was made after every three experimental samples, and the run was preceded by 5 QC conditioning injections to equilibrate the system. Solvent blanks (extraction solvent processed without tissue) were injected at the beginning, middle and end of the sequence to identify background and carry-over features.

### 2.3. Data Processing, Quality Control and Metabolite Annotation

Raw files were processed in Compound Discoverer 3.3 (Thermo Fisher Scientific) for peak detection, retention-time (RT) alignment, deconvolution, gap filling and area integration. Processing tolerances were: precursor mass tolerance 5 ppm, fragment mass tolerance 10 ppm, RT alignment window 0.2 min, signal-to-noise threshold 3, minimum peak intensity 1 × 10^5^.

System stability was assessed before downstream analysis by visual inspection of the overlaid total ion chromatograms (TIC) of QC injections for retention-time and intensity reproducibility, evaluation of peak capacity and signal drift across the analytical sequence, and calculation of the relative standard deviation (RSD) of each feature across QC injections. Features with QC RSD > 30% were discarded, and unsupervised principal component analysis (PCA) was used to confirm tight clustering of QC samples relative to the biological groups.

Annotation was performed by matching accurate precursor mass, isotope pattern and experimental MS/MS spectra against the Personalbio in-house authentic-standard library and the public libraries mzCloud, HMDB, KEGG, MassBank and METLIN. Acceptance criteria were: precursor mass error ≤ 5 ppm, isotope-pattern fit ≥ 70%, and mzCloud/library spectral match score ≥ 80 (best match, forward search). Because the great majority of annotations are at levels 2–3, isomeric ambiguity cannot be excluded, and all compound names below should be read as putative unless explicitly stated otherwise. Table S1 additionally reports, for every feature, the experimental MS1 *m*/*z*, mass error (ppm), ionization mode, adduct, RT in a representative QC injection, library source of the match, spectral match score, the assigned chemical class, and the raw and normalized peak area in each of the nine samples plus the QC injections. In particular, “alpha-berbine” corresponds to a protoberberine/tetrahydroprotoberberine-type alkaloid skeleton and is hereafter referred to as a tetrahydroprotoberberine-type alkaloid.

Annotated metabolites were screened for documented pharmacological relevance to *C. barometz* and to its traditional indications. A metabolite was included in the “medicinal fraction” when its structural class is reported as a bioactive constituent of *C. barometz* or of related ferns, or the individual compound has published bioactivity relevant to the traditional use of Gouji (antioxidant, anti-inflammatory, osteogenic, anti-osteoporotic, analgesic or haemostatic). Assignments were made from the compound annotations against the literature cited in the Introduction together with KEGG compound classification, and each retained metabolite was allocated to exactly one of nine mutually exclusive classes: organic acids, alkaloids, phenolic acids, coumarins/lignans, terpenoids, flavonoids, nucleosides, pterosins and organosulfur compounds. Compounds annotated as exogenous or process-derived were excluded from the medicinal fraction. For each sample the medicinal fraction was calculated as the summed relative abundance (% of total annotated signal) of all assigned metabolites, and class-level abundances were obtained by summing within class; values are reported as mean ± s.d. of three biological replicates together with the ratio to WP. Class-level differences between each substitute and WP were expressed as log_2_ fold change in the group means and tested by two-sided Student’s *t*-test on the per-replicate class sums (*p* < 0.05). For transparency and reproducibility, the complete list of included metabolites, assigned classes, annotation confidence and exclusion reasons is provided in Table S1.

### 2.4. Statistical Analysis and Visualization

Normalized peak areas were log2-transformed and Pareto-scaled before multivariate analysis. All multivariate and distance analyses were performed on the nine individual biological samples; group means are used for display only. Unsupervised PCA of the nine samples, with the pooled QC injections projected into the same space, was used to visualize overall metabolome structure and analytical stability. No supervised classification model was fitted, because three replicates per group cannot support a validated discriminant model; all group-level inferences therefore rest on univariate tests and on distance measures with explicit uncertainty.

Chemical distance to wild material was quantified at the replicate level. For each DM and ZP sample the distance to the WP group centroid was computed, and for each WP sample the distance to the centroid of the two remaining WP replicates was computed in a leave-one-out manner to provide a within-group reference. Five metrics were used: 1 − Pearson r and 1 − Spearman ρ (profile shape), Euclidean and Manhattan distance on log2-transformed intensities (magnitude, including low-abundance features) and Bray–Curtis dissimilarity on relative abundances (weighted towards dominant constituents). Distances are reported as mean ± s.d. of three replicates and compared between DM and ZP by two-sided Welch’s *t*-test. Given *n* = 3 per group, these comparisons have low statistical power and are reported as exploratory. A single criterion was applied to every differential-metabolite result reported in the text and figures: |log2 fold change| ≥ 1, two-sided Student’s *t*-test on log2-transformed normalized areas, and Benjamini–Hochberg false-discovery rate (FDR) < 0.05. Class-level comparisons used the same test applied to per-replicate class sums, with Benjamini–Hochberg correction across the nine classes; both raw and adjusted *p* values are reported. No other filter was used anywhere in the manuscript, and every figure legend restates the criterion applied in the corresponding panel.

Peak detection, QC evaluation, PCA and differential screening were performed on the Genes Cloud platform (Shanghai Personal Biotechnology Co., Ltd., Shanghai, China; https://www.genescloud.cn, accessed on 14 July 2026). Distance calculations, statistical testing and figure preparation used custom R scripts (R v4.3.1). Unless stated otherwise, data are mean ± standard deviation of three biological replicates.

## 3. Results

### 3.1. Overall Metabolite Profiling of the Three Cibotium-Related Materials

Analytical performance was assessed before any comparative analysis. Pooled quality control (QC) samples, prepared by combining equal aliquots of all nine biological samples, were injected at the beginning of the run and after every three study samples. The overlaid total ion chromatograms (TICs) of the QC injections were highly consistent in both peak shape and retention time in positive (Figure S1) and negative (Figure S2) ionization modes, indicating stable chromatographic and mass-spectrometric performance without detectable signal drift or carry-over.

**Figure 1:**
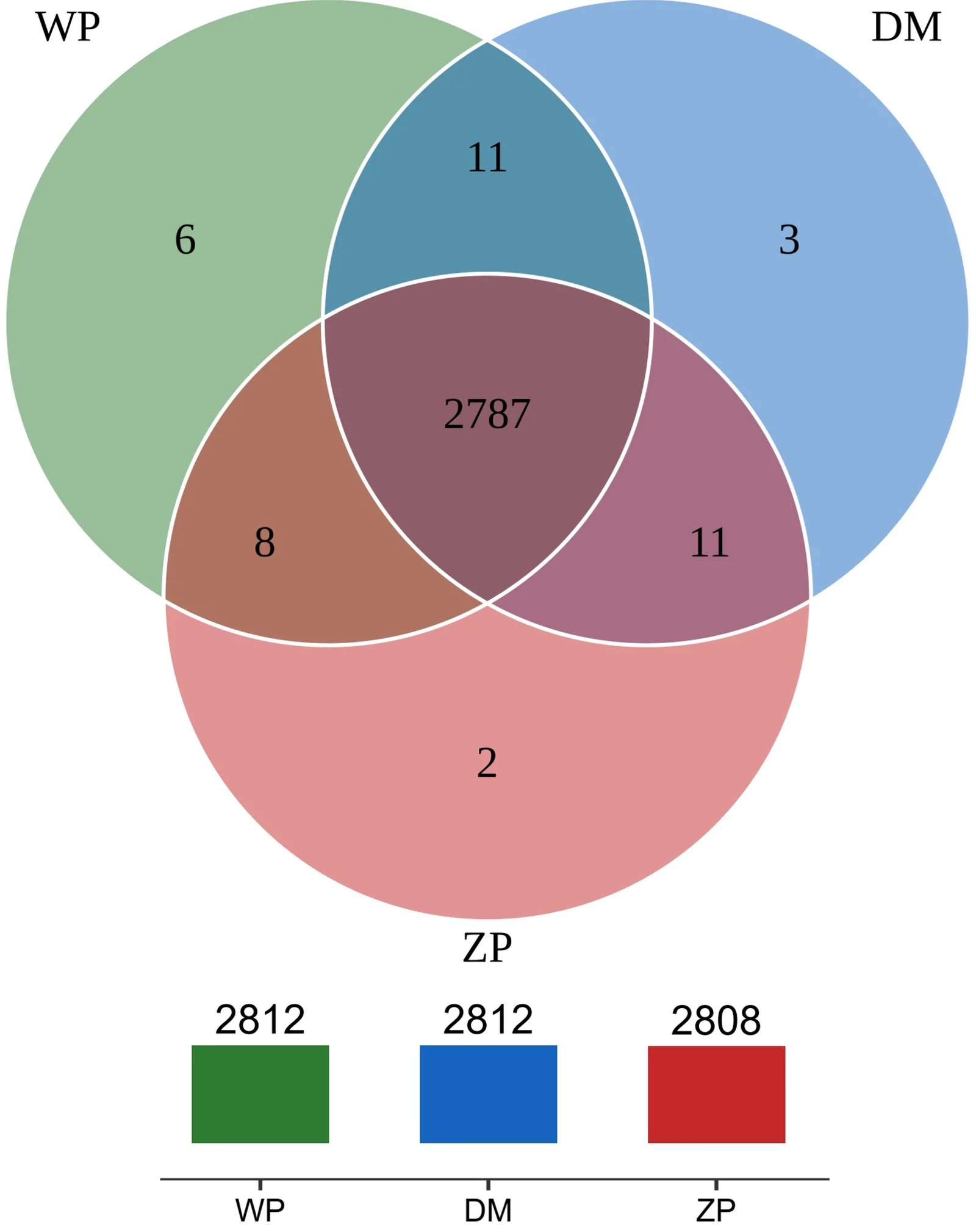
Overlap of annotated metabolite features detected in wild-collected (WP), field-domesticated (DM), and tissue-culture-derived (ZP) rhizomes of *Cibotium barometz*. Numbers in the Venn diagram indicate features meeting the predefined detection criterion in one or more production systems before missing-value imputation. Bars below the diagram show the total number of detected annotated features in each group. Presence or absence close to the detection threshold should not be interpreted as definitive biochemical gain or loss.

Untargeted metabolomic profiling of the wild-type rhizome (WP) and the two candidate substitutes (DM and ZP) yielded a total of 2828 non-redundant annotated metabolites across all samples, with 2812, 2812 and 2808 features detected in WP, DM and ZP, respectively (Figure 1). The three materials shared an overwhelming majority of their chemical space: 2787 metabolites (98.6% of the union) were common to all three groups, whereas group-specific metabolites were rare (WP, 6; DM, 3; and ZP, 2). Pairwise-exclusive overlaps were similarly small (WP ∩ DM, 11; DM ∩ ZP, 11; and WP ∩ ZP, 8). Thus, at the level of qualitative composition, the three materials are essentially equivalent, and differences between them are driven almost entirely by quantitative variation in shared metabolites rather than by the presence/absence of distinct compounds.

**Figure 2:**
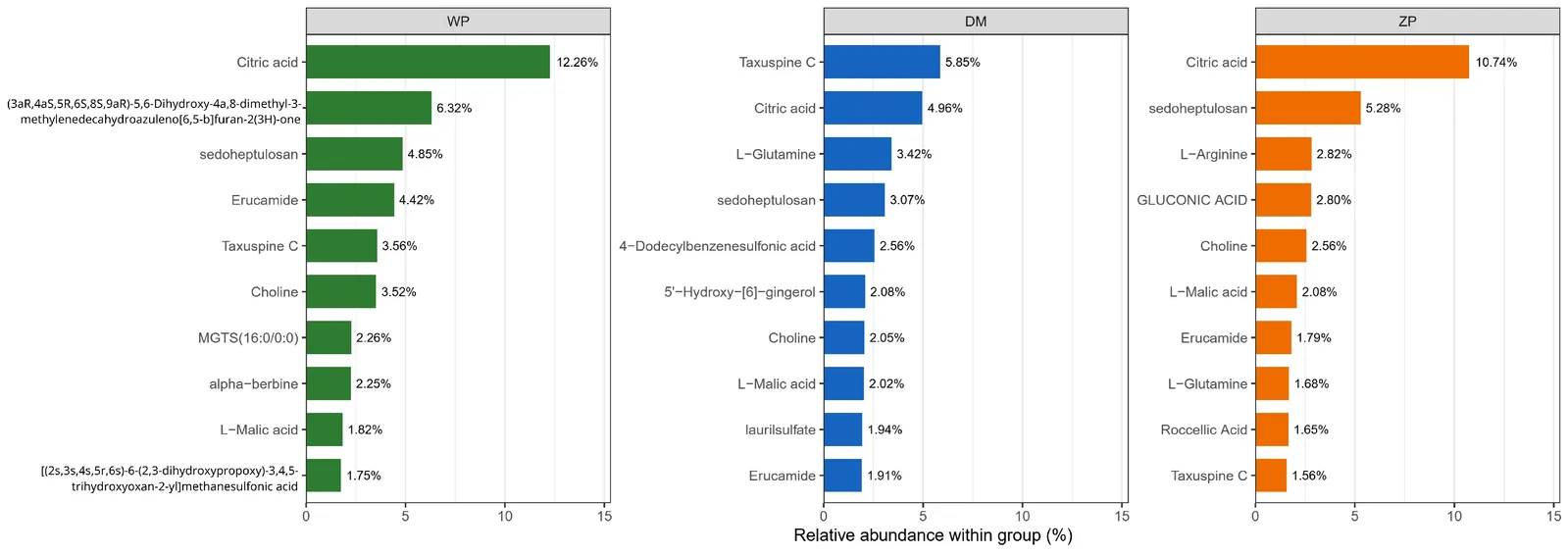
Annotated metabolites producing the ten highest normalized LC–MS peak-area responses in wild-collected (WP), field-domesticated (DM), and tissue-culture-derived (ZP) rhizomes of *Cibotium barometz*. Bars show the mean relative LC–MS response of each annotation as a percentage of the total annotated response within the corresponding group. These values are based on peak areas and do not represent absolute concentrations. Possible exogenous or process-derived annotations are shown for transparency but are not interpreted as endogenous medicinal constituents.

The most abundant constituents were also broadly conserved (Figure 2). Citric acid was the single dominant metabolite in WP (12.26% of the total signal) and in ZP (10.74%), but ranked only second in DM (4.96%). Sedoheptulosan, choline, L-malic acid, erucamide and taxuspine C were among the ten most abundant constituents of all three groups, albeit at different ranks and levels. Group-specific features of the abundance hierarchy included the large contribution of a WP-enriched guaianolide-type sesquiterpene lactone ((3aR,4aS,5R,6S,8S,9aR)-5,6-dihydroxy-4a,8-dimethyl-3-methylenedecahydroazuleno[6,5-b]furan-2(3H)-one; 6.32%) and of alpha-berbine (2.25%) in WP; the predominance of taxuspine C (5.85%), L-glutamine (3.42%) and surfactant-type signals (4-dodecylbenzenesulfonic acid, laurilsulfate) in DM; and the enrichment of gluconic acid (2.80%), L-arginine (2.82%) and roccellic acid (1.65%) in ZP. Together, these data indicate a common metabolic backbone dominated by organic acids, sugar-related compounds and quaternary amines, on which group-specific quantitative signatures are superimposed.

### 3.2. Global Metabolic Relatedness of DM and ZP to the Authentic Material WP

**Figure 3:**
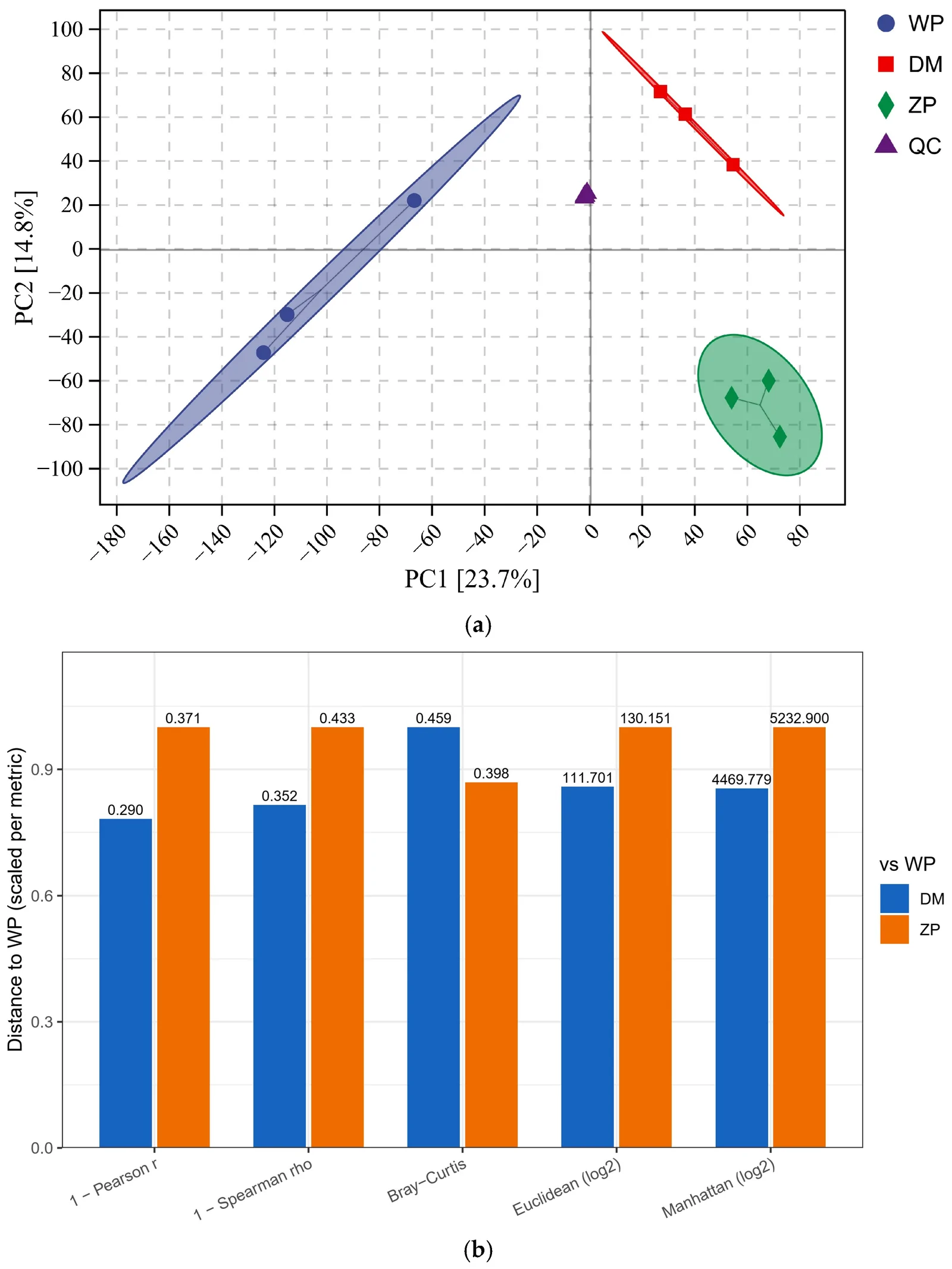

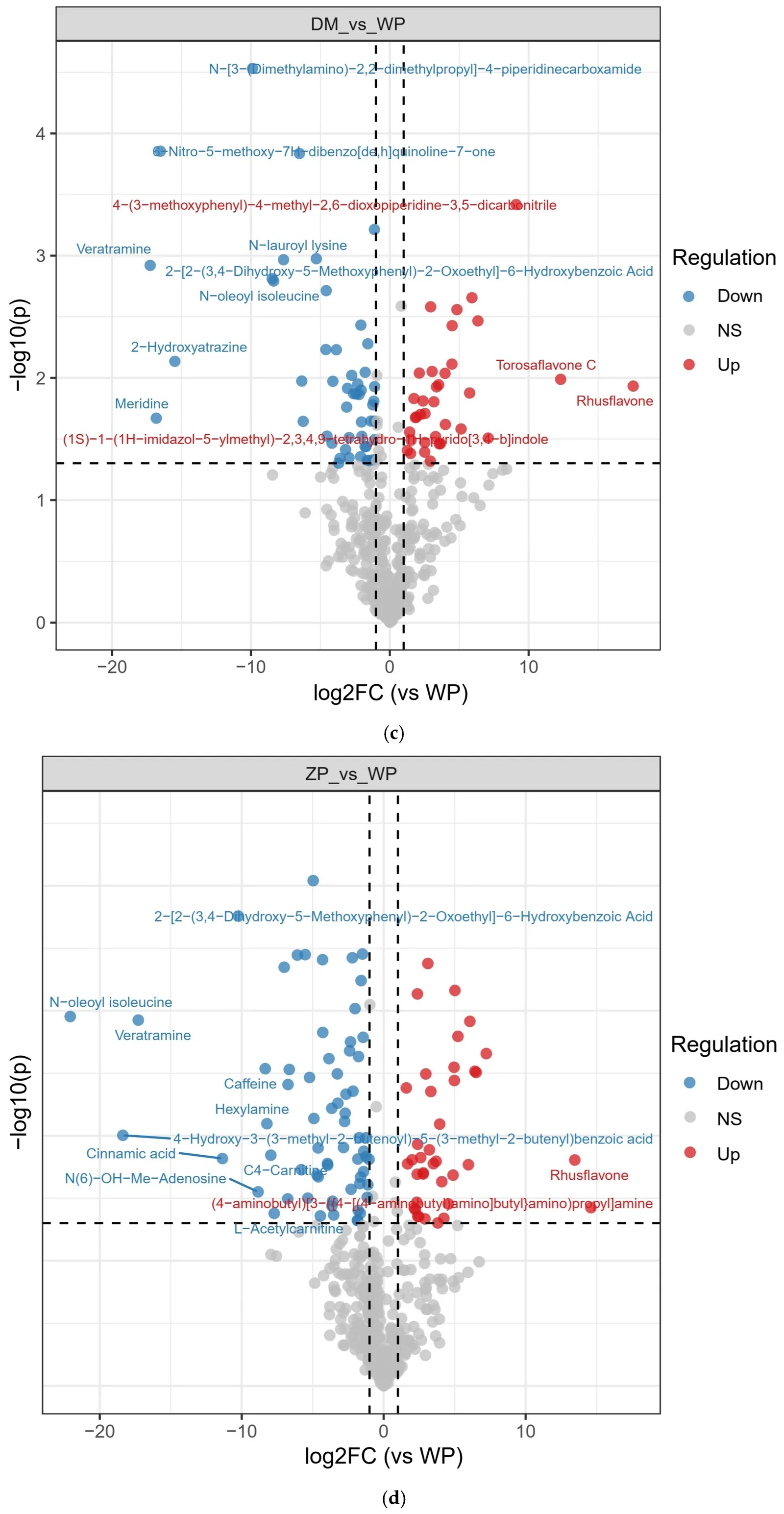
Metabolic relatedness of domesticated (DM) and tissue-cultured (ZP) rhizomes to wild-collected material (WP) of *C. barometz*. (**a**) PCA of the nine individual biological samples; each point is one replicate; ellipses denote 95% confidence regions. (**b**) Distance of each DM (blue) and ZP (orange) replicate to the WP centroid under five metrics; points are individual samples; bars are group means and error bars s.d.; each metric is plotted on its own axis because the metrics are not on a common numerical scale; *p* values are from two-sided Welch’s *t*-tests (DM vs. ZP). (**c**,**d**) Volcano plots for DM versus WP (**c**) and ZP versus WP (**d**). Red, increased; blue, decreased (|log_2_FC| ≥ 1 and Benjamini–Hochberg FDR < 0.05); grey, not significant. Dashed lines mark the fold-change and significance thresholds; representative annotated metabolites are labelled.

To determine which candidate is chemically closer to WP, group-mean profiles were compared by principal component analysis (PCA) and by five complementary distance metrics (Figure 3). Principal component analysis (PCA) was performed on the log_2_-transformed, Pareto-scaled peak-area matrix of all nine biological samples (three per group) together with the pooled QC injections, rather than on group means, so that within-group variation could be visualized. The first two principal components explained 23.7% (PC1) and 14.8% (PC2) of the total variance (38.5% cumulative), a modest proportion that is typical of untargeted profiling datasets in which several thousand features contribute small, largely independent fractions of variance. Despite this, the three sample types occupied clearly separated regions of the score plot (Figure 3a): WP samples were positioned at negative PC1 values, DM samples at positive PC1 and positive PC2, and ZP samples at positive PC1 and negative PC2, with no overlap between groups. The pooled QC injections clustered tightly near the origin and were markedly more compact than any biological group, confirming that analytical variation was small relative to the biological differences between wild-collected, field-domesticated and tissue-cultured rhizomes.

The direction of the difference, however, depended on how distance was defined (Figure 3b). Correlation-based metrics, which are dominated by the overall shape of the profile across all 2828 metabolites, ranked DM closer to WP than ZP (1 − Pearson r: 0.290 vs. 0.371; 1 − Spearman ρ: 0.352 vs. 0.433), and magnitude-based metrics on log_2_-transformed data gave the same ordering (Euclidean: 111.701 vs. 130.151; Manhattan: 4469.779 vs. 5232.900). By contrast, Bray–Curtis dissimilarity, which is computed on relative abundances and is therefore weighted towards the few highly abundant constituents, ranked ZP closer to WP (0.398 vs. 0.459). This apparent discrepancy is readily explained by the abundance data in Figure 2: ZP reproduces the WP-like dominance of citric acid and other major organic acids, thereby minimizing abundance-weighted dissimilarity, whereas DM better reproduces the rank order and breadth of the WP profile across the full metabolite set. Four of the five metrics, including all those computed on the complete profile, thus support DM as the globally closer analogue of WP, while ZP is the closer match with respect to the quantitatively dominant constituents.

Differential analysis against WP was consistent with the moderate but genuine divergence of both candidates. DM_vs_WP yielded 610 differentially accumulated metabolites (284 up, 326 down; Figure 3c), with the strongest depletions in WP-characteristic alkaloids and acyl-amino acids (*N*-[3-(dimethylamino)-2,2-dimethylpropyl]-4-piperidinecarboxamide, 6-nitro-5-methoxy-7H-dibenzo[de,h]quinoline-7-one, veratramine, meridine, *N*-lauroyl lysine, *N*-oleoyl isoleucine) and the strongest enrichments in biflavonoids (rhusflavone and torosaflavone C). ZP_vs_WP yielded 821 differentially accumulated metabolites (353 up, 468 down; Figure 3d), again dominated by depletion of *N*-oleoyl isoleucine, veratramine, cinnamic acid, caffeine, carnitines and N(6)-OH-Me-adenosine, and by marked accumulation of rhusflavone and polyamines. Fold changes in this magnitude arise where the WP signal lies at or below the detection limit and has been gap-filled, and they should therefore be regarded as qualitative indications of enrichment rather than quantitative estimates. The shared depletion of veratramine, *N*-oleoyl isoleucine and 2-[2-(3,4-dihydroxy-5-methoxyphenyl)-2-oxoethyl]-6-hydroxybenzoic acid in both candidates identifies a small set of WP-preferential markers that may be useful for authentication.

### 3.3. Composition and Differential Accumulation of Medicinally Relevant Constituents

**Figure 4:**
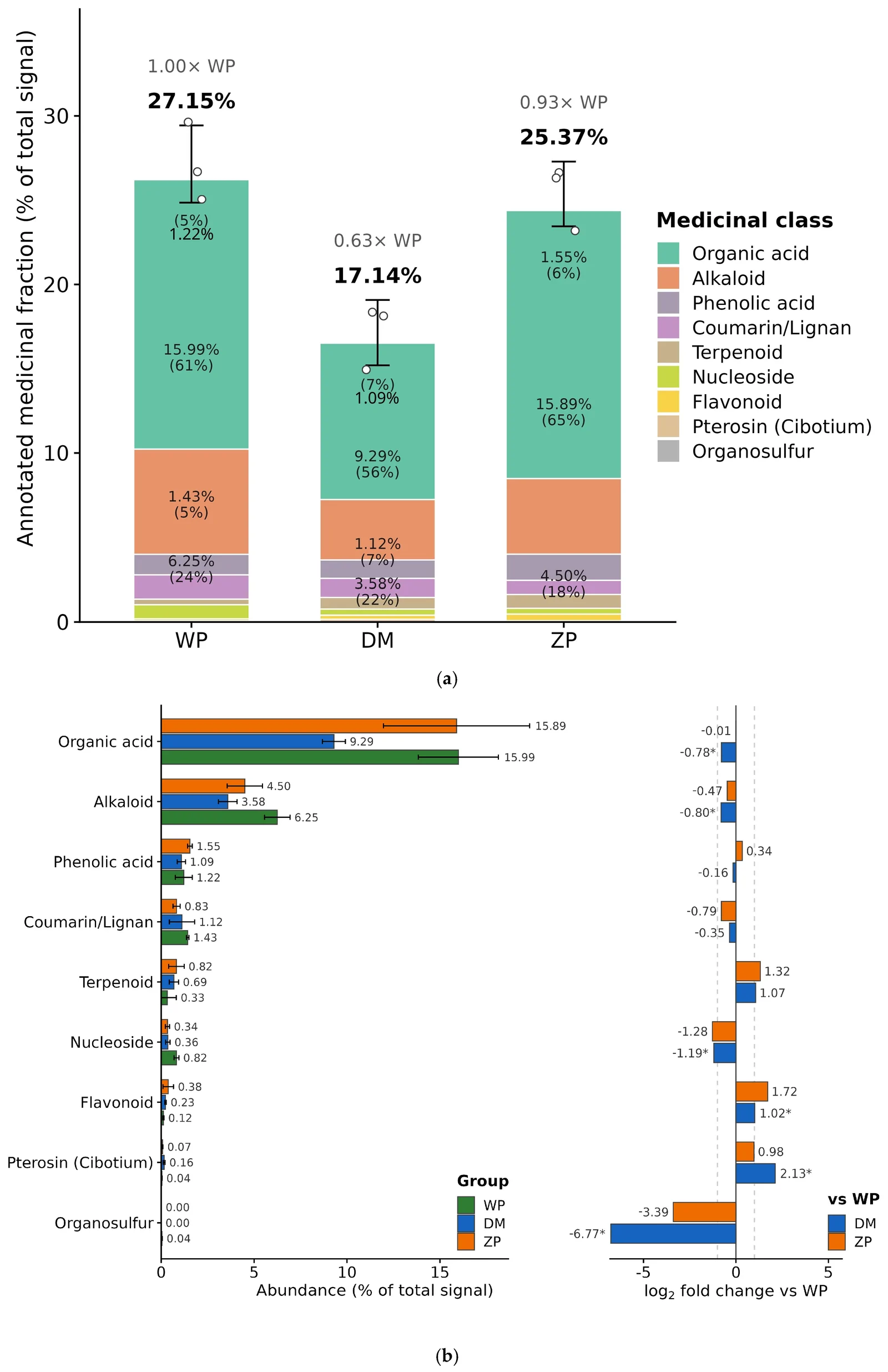
Composition and differential accumulation of medicinally relevant metabolite classes. (**a**) Stacked bars showing the annotated medicinal fraction (percentage of total signal) in WP, DM and ZP, partitioned into nine classes. Bold numbers above each bar give the total medicinal fraction, and grey numbers give the ratio relative to WP. Values within the bars give the absolute contribution of the major classes and, in parentheses, their share of the medicinal fraction. Error bars represent s.d.; open circles denote individual biological replicates. (**b**) Left: class-level abundance (% of total signal, mean ± s.d.) in WP (green), DM (blue) and ZP (orange). Right: log2 fold change in each class in DM and ZP relative to WP; dashed lines mark |log_2_FC| = 1; asterisks indicate Benjamini–Hochberg-adjusted *p* < 0.05 (two-sided Student’s *t*-test on per-replicate class sums).

Metabolites with documented pharmacological relevance were annotated and assigned to nine classes (Figure 4). The annotated medicinal fraction accounted for 27.15% of the total signal in WP, 25.37% in ZP (0.93× WP) and 17.14% in DM (0.63× WP) (Figure 4a). In all three materials the medicinal fraction was dominated by organic acids (WP 15.99%, 61% of the medicinal fraction; DM 9.29%, 56%; and ZP 15.89%, 65%), followed by alkaloids (WP 6.25%, 24%; DM 3.58%, 22%; and ZP 4.50%, 18%), phenolic acids (1.09–1.55%) and coumarins/lignans (0.83–1.43%). Terpenoids, nucleosides, flavonoids, *Cibotium*-characteristic pterosins and organosulfur compounds each contributed less than 1% of the total signal. ZP therefore reproduced the medicinal profile of WP most closely in terms of total burden and organic-acid content, whereas DM retained only about two-thirds of the WP medicinal signal.

Class-level comparison with WP (Figure 4b) refined this picture. In DM, organic acids (log_2_FC = −0.78, *p* < 0.05), alkaloids (−0.80, *p* < 0.05), nucleosides (−1.19, *p* < 0.05) and organosulfur compounds (−6.77, *p* < 0.05) were significantly reduced, while flavonoids (+1.02, *p* < 0.05) and pterosins (+2.13, *p* < 0.05) were significantly increased. In ZP, no class differed significantly from WP after correction, although terpenoids (+1.32), flavonoids (+1.72) and pterosins (+0.98) trended upward and nucleosides (−1.28), organosulfur compounds (−3.39), coumarins/lignans (−0.79) and alkaloids (−0.47) trended downward. Notably, organic acids in ZP were essentially indistinguishable from WP (log_2_FC = −0.01).

**Figure 5:**
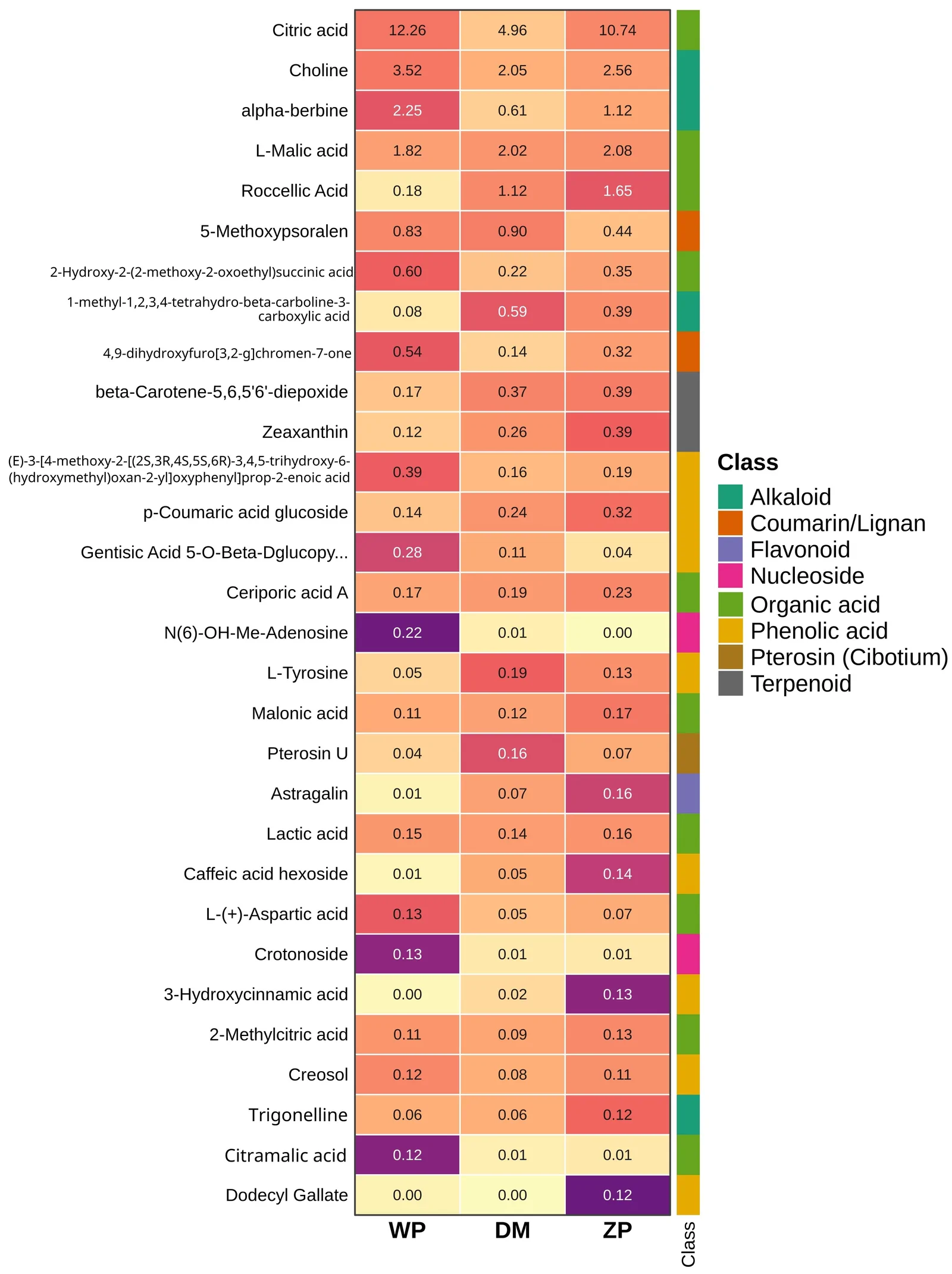
Heatmap of 30 high-response annotations in the literature-prioritized metabolite subset of wild-collected (WP), field-domesticated (DM), and tissue-culture-derived (ZP) rhizomes of *Cibotium barometz*. Cell values show mean relative LC–MS peak-area responses rather than absolute concentrations. The colour scale represents relative variation within each metabolite row. The annotation bar indicates the assigned structural class. Compound names represent putative annotations unless confirmed with authentic standards.

At the level of individual markers (Figure 5), the 30 most abundant annotated compounds fell into three groups. The first comprises compounds conserved across all three materials, including citric acid, L-malic acid, choline, lactic acid and malonic acid, which maintained comparable relative abundances (e.g., L-malic acid, 1.82/2.02/2.08% in WP/DM/ZP). The second comprises WP-preferential compounds, notably alpha-berbine (2.25 vs. 0.61 and 1.12%), N(6)-OH-Me-adenosine (0.22 vs. 0.01 and 0.00%), crotonoside (0.13 vs. 0.01 and 0.01%), citramalic acid (0.12 vs. 0.01 and 0.01%), gentisic acid 5-O-β-D-glucopyranoside and 4,9-dihydroxyfuro[3,2-g]chromene derivatives, all of which were depleted in both substitutes and therefore represent candidate discriminating markers of authentic material. The third comprises substitute-enriched compounds: roccellic acid, 1-methyl-1,2,3,4-tetrahydro-β-carboline, L-tyrosine and pterosin U in DM, and astragalin, caffeic acid hexoside, 3-hydroxycinnamic acid, dodecyl gallate, trigonelline and the carotenoids zeaxanthin and β-carotene-5,6,5′,6′-diepoxide in ZP. Overall, ZP preserves the quantitative medicinal profile of WP more faithfully, in particular, its organic-acid-dominated core, whereas DM shows a systematic reduction in total medicinal signal accompanied by a relative shift towards flavonoids and pterosins.

## 4. Discussion

*Cibotium barometz* is harvested almost exclusively as a wild rhizome, and the CITES-listed status of the genus makes the chemical validation of propagated material a prerequisite rather than an afterthought for any sustainable supply strategy. The present study addresses two linked questions: which constituents of the rhizome actually carry the pharmacological identity of the drug, and to what extent tissue-cultured (ZP) and domesticated (DM) plants reconstitute that identity relative to wild-type material (WP). The answer that emerges is encouraging in its qualitative dimension and instructive in its quantitative one.

The most robust finding is that neither propagation route alters the chemical repertoire of the rhizome in any categorical sense. Of the 2828 annotated metabolites, 2787 (98.6%) were detected in all three materials, and group-exclusive features numbered fewer than ten per group—a count that is within the range expected from detection-limit effects around low-abundance features rather than from genuine biosynthetic gain or loss. This is a biologically meaningful result: the enzymatic machinery of the rhizome, including the pathways generating the *Cibotium*-characteristic pterosins, phenolic acids and alkaloids [11], remains intact under both in vitro regeneration and field domestication. Whatever differences exist between wild and propagated material are therefore matters of flux and allocation, which are in principle tractable through agronomic optimization, rather than matters of pathway integrity, which would not be. The corollary for quality control is that presence/absence fingerprinting cannot distinguish these materials; only quantitative, abundance-aware criteria can.

When these quantitative criteria are applied, the ranking of the two candidates depends on the aspect of the metabolome that each metric emphasizes [15,16]. Metrics computed across the full profile, whether correlation-based (1 − Pearson, 1 − Spearman) or magnitude-based on log_2_-transformed intensities (Euclidean, Manhattan), all placed DM closer to WP. Bray–Curtis dissimilarity, computed on relative abundances and therefore dominated by the handful of constituents that account for most of the signal, reversed the ranking in favour of ZP. These results are complementary rather than contradictory. Log-transformed, all-feature metrics weight the long tail of minor metabolites and thus describe the overall architecture of the metabolome, whereas abundance-weighted metrics describe the bulk chemical composition that a decoction, and hence a patient, actually receives. Their disagreement indicates that DM and ZP diverge from WP along largely independent axes, as the PCA also showed: PC1 mainly separated wild-collected from cultivated material, whereas PC2 distinguished field-domesticated from tissue-cultured rhizomes, indicating that growth environment and propagation route are both imprinted on the metabolite profile.

The pharmacologically weighted analysis resolves this ambiguity in a way that is directly relevant to medicinal use. Restricting attention to the nine annotated classes of medicinally relevant constituents, ZP retained 93% of the WP medicinal signal (25.37% vs. 27.15% of total signal) whereas DM retained only 63% (17.14%). The deficit in DM was not distributed evenly but concentrated in the two classes that dominate the medicinal fraction—organic acids and alkaloids (−0.80)—together with nucleosides (−1.19) and organosulfur compounds (−6.77). In ZP, by contrast, no class differed significantly from WP after correction, and organic acids were essentially superimposable on the wild-type value. Given that organic acids constitute 54–65% of the medicinal fraction in all three materials and that citric and malic acid dominate the total profile, this near-identity of the organic-acid core is the single strongest argument for the pharmacological interchangeability of tissue-cultured material [17,18]. The organic-acid pool of the *Cibotium* rhizome is not chemically exotic, but it is functionally relevant: citrate and malate contribute to the mineral-chelating and osteogenic-supportive properties attributed to the drug in the treatment of lumbar and joint disorders, and they govern the extraction behaviour of co-occurring phenolics and alkaloids in aqueous decoctions [19,20,21].

It is worth dwelling on the apparent paradox that the in vitro-regenerated material rather than the field-domesticated material better preserves the medicinal profile. Two non-exclusive explanations seem plausible. First, the DM plants, though grown ex situ, experience a soil, light and microbial environment that differs from the deeply shaded, humus-rich forest-floor habitat of wild *C. barometz*, and secondary-metabolite allocation in ferns is known to be strongly modulated by irradiance, water status and rhizosphere biota [22]; comparable reallocation has been documented in other cultivated medicinal species, in which relaxed biotic pressure and higher nutrient supply reduce nitrogen- and sulfur-containing defence metabolites [23,24]. We note that most of this evidence comes from angiosperm systems with glucosinolate or alkaloid chemistry that has no direct counterpart in ferns [25,26]. Second, tissue culture supplies a defined, sucrose-buffered, stress-free medium in which primary carbon metabolism—and hence the tricarboxylic-acid-derived organic acid pool—is maintained at a high and stable level, which may fortuitously reproduce the WP organic-acid signature. The compensating shift in DM towards flavonoids (log_2_FC = +1.02) and pterosins (+2.13), both of which are photoprotective and antiherbivore in function, supports the irradiance/stress interpretation and indicates that domesticated plants are not simply “poorer” but chemically re-tuned [27,28]. From an applied standpoint this is actionable: shade management, substrate composition and nitrogen regime are the obvious levers for restoring the alkaloid and organic-acid content of domesticated stock, and the fact that the biosynthetic capacity is demonstrably present makes such optimization a realistic goal.

Beyond the class-level comparison, the analysis identifies a small, coherent set of WP-preferential constituents that were depleted in both substitutes and therefore constitute candidate authentication markers [29,30]: veratramine, *N*-oleoyl isoleucine, 2-[2-(3,4-dihydroxy-5-methoxyphenyl)-2-oxoethyl]-6-hydroxybenzoic acid, and, among the abundant medicinal compounds, alpha-berbine (2.25% in WP vs. 0.61% and 1.12%), N(6)-hydroxymethyl-adenosine (0.22% vs. ≤0.01%), crotonoside and citramalic acid. The near-complete loss of the modified nucleosides in both propagated materials is striking and reproducible across metrics, and their high fold-change combined with low background makes them attractive targets for a targeted LC–MS/MS assay to discriminate wild from propagated rhizome—a capability with obvious relevance to CITES enforcement as well as to pharmacopoeial grading [1,3,7]. Conversely, both substitutes accumulated rhus flavone to an extreme degree, and ZP was enriched in astragalin, caffeic acid hexoside, dodecyl gallate, trigonelline and carotenoids, including zeaxanthin [31,32,33]. These substitute-enriched compounds are not liabilities per se—several are well-characterized antioxidants and biflavonoids with documented anti-inflammatory activity [34,35,36]—but they represent a genuine compositional novelty relative to the traditional drug and would need to be considered in any regulatory dossier proposing propagated material as a pharmacopoeial substitute.

Several constraints bound the conclusions. The design confounds propagation route with plant age and growing environment, and with three biological replicates per group from one site and one collection date it has limited power and no capacity to generalize across seasons or provenances. The study is chemical, not pharmacological: no bioassay was performed, and metabolite-level similarity is a proxy for, not a demonstration of, therapeutic equivalence. Untargeted LC–MS is semi-quantitative; class sums are response-weighted and do not represent chemical amounts, so statements such as “93% of the wild profile retained” refer to detected signal, not to delivered dose.

In conclusion, wild-collected, field-domesticated and tissue-cultured *C. barometz* rhizomes share essentially the same annotated metabolite repertoire, and differ quantitatively rather than qualitatively. Domesticated material reproduces the overall architecture of the wild metabolome more closely across the full feature set, whereas tissue-cultured material more closely reproduces the quantitatively dominant, putatively bioactive constituents, in particular, the organic-acid core. On chemical grounds alone, micropropagated material is therefore the closer analogue of wild rhizome with respect to the constituents that dominate an extract, while domesticated material shows a broad reduction in these constituents offset by increases in flavonoids and pterosins.

Future work should proceed along three lines. First, bioassay-guided comparison of the three materials in models relevant to the traditional indications—osteogenic differentiation, anti-inflammatory and hemostatic assays—to test whether the chemical differences reported here have functional consequences. Second, absolute quantification of the candidate markers and of the dominant classes against authentic standards by targeted LC–MS/MS or qNMR, replacing the semi-quantitative peak-area measures used here. Third, an age- and site-matched design, ideally with common-garden transplantation, to separate propagation route from plant age and growing environment, and multi-season, multi-provenance sampling with larger replication to establish marker stability. Only after chemical validation, exposure assessment, and biological comparison should the suitability of propagated rhizomes for pharmacopeia or clinical substitution be considered.

## Data Availability

The processed metabolomics data and associated metabolite annotation metadata supporting this study are available in Figshare at https://doi.org/10.6084/m9.figshare.33295689. The data relevant to the present manuscript comprise the WP\DM\ZP sample groups.

## Supplementary Materials

The following supporting information can be downloaded at: https://www.mdpi.com/article/10.3390/life16091435/s1, Table S1: The complete list of included metabolites for nine samples of *C. barometz.* Figure S1. Overlaid total ion chromatograms (TICs) of the four pooled quality control (QC) injections acquired in positive ionization mode. Each trace represents one QC injection distributed across the analytical sequence (QC1–QC4); the number after each QC label is the total ion count used to normalize that trace. Figure S2. Overlaid total ion chromatograms (TICs) of the four pooled QC injections acquired in negative ionization mode. Presentation and normalization are as described for Figure S1.
